# A bioinformatics-to-clinic sequential approach to analysis of prostate cancer biomarkers using TCGA datasets and clinical samples: a new method for precision oncology?

**DOI:** 10.18632/oncotarget.20448

**Published:** 2017-08-24

**Authors:** Hidekazu Yoshie, Anna S. Sedukhina, Kimino Minagawa, Keiko Oda, Shigeko Ohnuma, Nobuyuki Yanagisawa, Ichiro Maeda, Masayuki Takagi, Hiroya Kudo, Ryuto Nakazawa, Hideo Sasaki, Toshio Kumai, Tatsuya Chikaraishi, Ko Sato

**Affiliations:** ^1^ Department of Pharmacogenomics, St. Marianna University, Kawasaki, Japan; ^2^ Department of Urology, St. Marianna University, Kawasaki, Japan; ^3^ Department of Pathology, St. Marianna University, Kawasaki, Japan

**Keywords:** bioinformatics, precision oncology, prostate cancer, PEG10, neuroendocrine prostate cancer

## Abstract

Biomarker-driven cancer therapy has met with significant clinical success. Identification of a biomarker implicated in a malignant phenotype and linked to poor clinical outcome is required if we are to develop these types of therapies. A subset of prostate adenocarcinoma (PACa) cases are treatment-resistant, making them an attractive target for such an approach. To identify target molecules implicated in shorter survival of patients with PACa, we established a bioinformatics-to-clinic sequential analysis approach, beginning with 2-step *in silico* analysis of a TCGA dataset for localized PACa. The effect of candidate genes identified by *in silico* analysis on survival was then assessed using biopsy specimens taken at the time of initial diagnosis of localized and metastatic PACa. We identified PEG10 as a candidate biomarker. Data from clinical samples suggested that increased expression of PEG10 at the time of initial diagnosis was linked to shorter survival time. Interestingly, PEG10 overexpression also correlated with expression of chromogranin A and synaptophysin, markers for neuroendocrine prostate cancer, a type of treatment-resistant prostate cancer. These results indicate that PEG10 is a novel biomarker for shorter survival of patients with PACa. Also, PEG10 expression at the time of initial diagnosis may predict focal neuroendocrine differentiation of PACa. Thus, PEG10 may be an attractive target for biomarker-driven cancer therapy. Thus, bioinformatics-to-clinic sequential analysis is a valid tool for identifying targets for precision oncology.

## INTRODUCTION

Precision oncology, also called biomarker-driven therapy, has greatly improved clinical outcomes in recent years. Biomarker-driven therapies such as trastuzumab for HER2-positive breast cancer and imatinib for chronic myeloid leukemia [1, 2] highlight the efficacy of targeting biomarkers associated with a poor prognosis and illustrate the importance of identifying those biomarkers involved in poor clinical outcomes for malignancies.

Prostate adenocarcinoma (PACa) is one of the most common cancers in men. In the U.S., the 5 year relative survival rate of early stage PACa is >99% [3]; yet PACa is the second leading cause of cancer-related death in the U.S. This suggests that a subset of PACa is treatment-resistant [3]. To determine the prognosis of a PACa patient, physicians measure levels of prostate-specific antigen (PSA), and use clinical staging and the Gleason score, which is a grading system based on the architectural pattern of tissue from a PACa biopsy [4-6]. In addition to these factors, castration-resistant prostate cancer (CRPC), a transformed prostate cancer mainly caused by continuous androgen deprivation, is linked to a poor prognosis due to limited therapeutic options [7]. Neuroendocrine prostate cancer is a class of CRPC showing neuroendocrine differentiation [8]. Because of its poor prognosis and treatment resistance, there is significant unmet need for new neuroendocrine prostate cancer treatments. However, the best molecular targets for neuroendocrine prostate cancer therapies have not yet been identified [8].

To identify biomarkers of poor prognosis in PACa, we developed a bioinformatics-to-clinic sequential analysis approach and used it to identify a candidate biomarker linked to shorter survival. To evaluate the predictive power of our analytical approach, we examined biopsy samples to see if expression of our identified gene, PEG10, at the time of initial diagnosis affected clinical outcome. PEG10 was also of interest because a recent report implicates it in neuroendocrine differentiation of PACa [9]. Therefore, we also investigated the link between PEG10 expression and neuroendocrine differentiation in clinical samples.

## RESULTS

### Overview of the bioinformatics-to-clinic sequential analysis method

**Figure 1:**
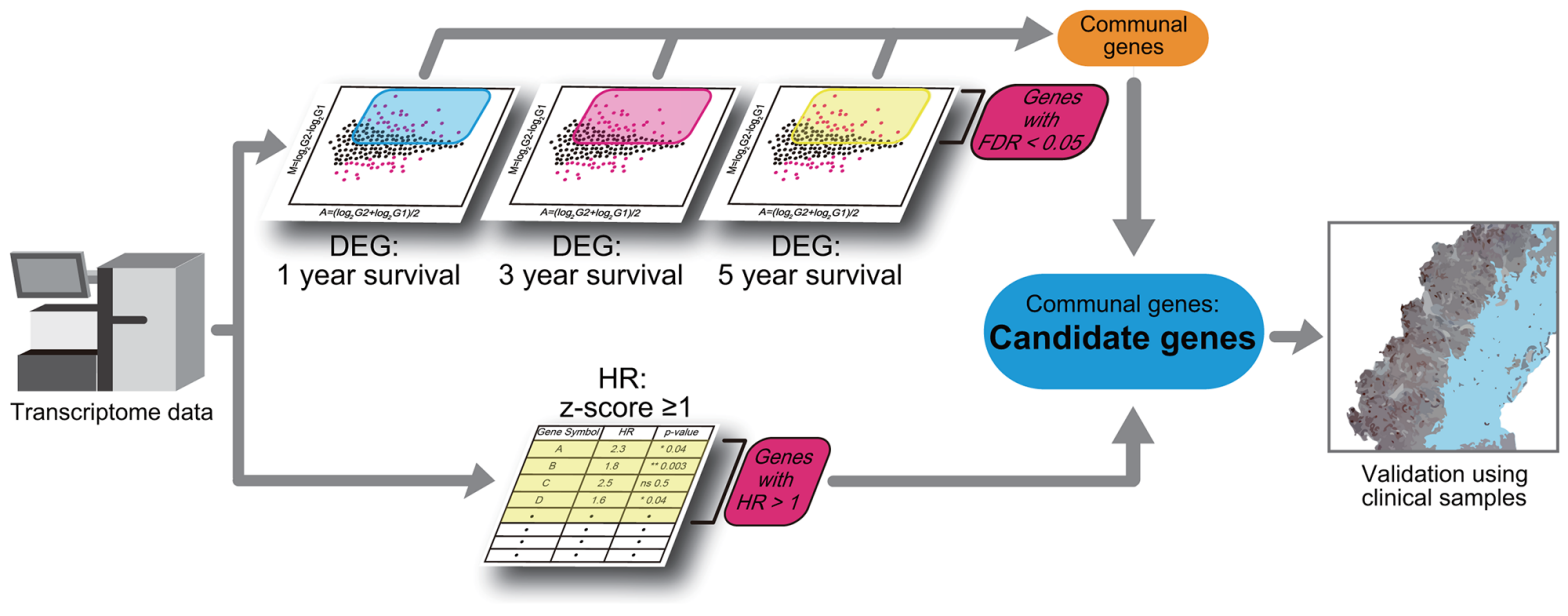
Schematic showing the bioinformatics-to-clinic sequential analysis method Bioinformatics-to-clinic sequential analysis from transcriptome data.

To identify biomarkers that predict shorter survival of patients with PACa, we used two analytical procedures based on the TCGA dataset for localized PACa, followed by validation in clinical samples (Table 1). First, we extracted PACa cases with a Gleason score ≥8; this is because higher Gleason scores are linked to poor clinical outcomes [4]. Next, differentially expressed genes (DEGs) were identified based on relapse-free survival (RFS). DEGs that were highly expressed in the cohort with shorter survival were extracted. Second, the RFS hazard ratio (HR) for all overexpressed genes was calculated to identify genes linked to shorter RFS. Genes identified in both analyses were designated final candidate genes. Finally, the effect of the candidate gene expression on survival was validated using clinical samples (Figure 1).

**Table 1:** Baseline characteristics of TCGA database (Gleason score > 8)

Total cases	Total	1y		3y		5y	
		<1	≥1	<3	≥3	<5	≥5
	201	24	102	48	45	57	15
Age							
Average	62.4	63.3	62.3	62.7	62.3	62.4	61.5
Range	44-78	53-72	44-76	46-78	46-71	46-78	46-70
T category							
T2b	3		2		1		1
T2c	24	1	17	3	8	3	3
T3a	62	5	31	14	12	18	5
T3b	103	16	47	29	22	34	6
T4	7	2	3	2	2	2	
NA	2		2				
Gleason score							
3+5	7	1	6	1	3	1	2
4+4	50	3	33	9	10	10	3
5+3	7		6		4		1
4+5	97	13	40	26	23	34	8
5+4	37	7	15	11	4	11	1
5+5	3		2	1	1	1	
Tissue							
Prostate	201	24	102	48	45	57	15
Histology							
Adenocarcinoma	145	17	72	38	30	43	11
Aca mixed subtype	2	1	1	1		1	
Mucinous ca	1						
Signet ring cell ca	1		1		1		
Infiltrating duct ca	8	1	3	2	1	2	1
Acinar cell ca	44	5	25	7	13	6	3

Adenocarcinoma: Adenocarcinoma NOS

Aca: Adenocarcinoma

Ca: carcinoma

### Identification of DEGs

**Figure 2:**
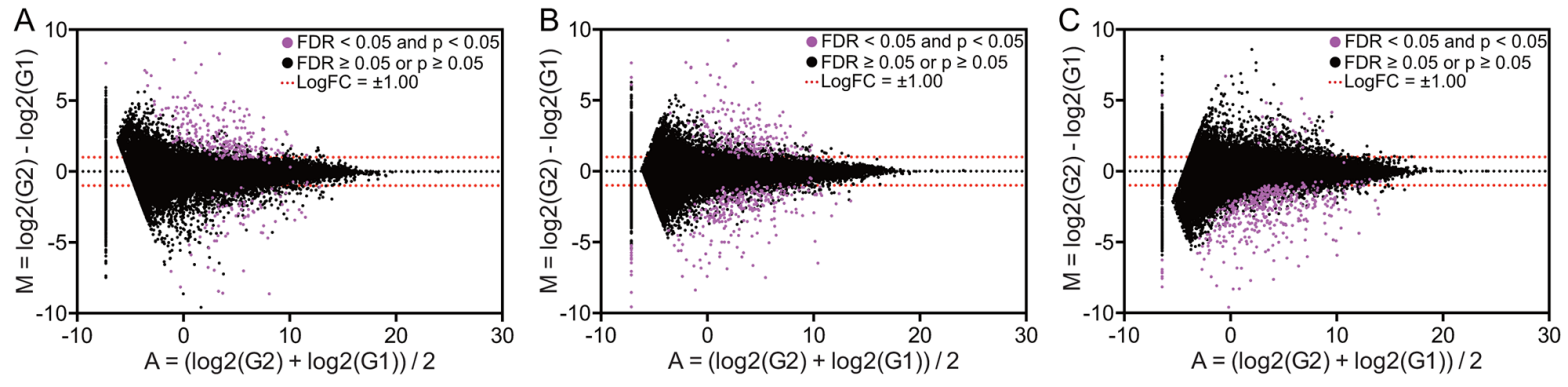
Alterations in gene expression in patients with different prognoses MA plot showing gene expression in three different settings: relapse-free survival at 1 year **(A)**, 3 years **(B)**, or 5 years **(C)**. Pink dots denote differentially expressed genes (defined as FDR <0.05). G1 and G2 indicate cohorts with longer and shorter life spans, respectively.

The TCGA dataset was divided into groups based on RFS at 1, 3, or 5 years (Figure 1, Table 1, and Supplementary Table 1). The number of cases in the shorter/longer survival cohorts was 24/102 (1 year), 48/45 (3 year), and 57/15 (5 year) (Table 1 and Supplementary Table 1). We then identified DEGs (false discovery rate (FDR) <0.05) that were up-regulated (logFC ≥1) (Figure 2). The number of up-regulated DEGs was 233 (1 year), 195 (3 year), and 29 (5 year). Eight DEGs overlapped across these time points (Table 2).

**Table 2:** Differentially expressed genes overlapped

Gene symbol	1 year		3 year		5 year	
	LogFC	FDR	LogFC	FDR	LogFC	FDR
CDH17	1.60	1.82E-02	1.97	5.29E-04	2.86	1.45E-02
CDH20	4.20	1.01E-26	3.14	9.04E-07	3.63	1.39E-02
CHRNB2	3.08	1.94E-13	2.72	2.97E-06	2.94	4.58E-02
ELAVL3	2.40	5.03E-08	1.64	2.71E-02	2.99	1.48E-02
GDAP1L1	3.16	6.78E-16	2.75	2.19E-06	2.96	4.34E-02
GRIA4	3.91	3.37E-12	4.78	2.11E-08	4.78	2.59E-02
PEG10	2.80	4.62E-13	2.52	2.06E-06	2.86	2.18E-02
SYT5	3.08	1.72E-08	3.24	2.22E-06	3.66	3.37E-02

FDR: false discovery rate

LogFC: logarithmic fold change

### Calculation of the RFS HR

We calculated the RFS HR for each gene that was overexpressed (z-score ≥1) using a Cox proportional hazard model. Before calculating the HR, the number of cases showing overexpression of each gene was examined. If a gene was overexpressed in less than 5% of the total cohort, it was excluded from further analysis because a HR calculated from a disproportionate distribution is not reliable. Thus, 2527 genes (13.3% of the identified genes) were excluded. Next, we identified genes responsible for shorter RFS (HR >1) among genes with a significant p-value for RFS HR (Figure 1 and Table 3). The number of genes responsible for shorter RFS was 630.

**Table 3:** Top 40 genes affecting hazard ratio

Gene symbol	HR (95%CI)	p-value
NRN1L	4.468 (2.085 – 9.574)	1.18E-04
NDUFB11	4.304 (1.785 – 10.38)	1.16E-03
ING4	4.034 (2.217 – 7.337)	4.91E-06
ZBTB8B	4.022 (1.823 – 8.875)	5.66E-04
GPR35	3.993 (1.826 – 8.731)	5.22E-04
B4GALT2	3.912 (1.797 – 8.52)	5.92E-04
C14ORF178	3.881 (1.838 – 8.193)	3.75E-04
SARS	3.761 (1.868 – 7.574)	2.08E-04
ACADVL	3.759 (1.744 – 8.103)	7.26E-04
RPS29	3.725 (1.567 – 8.852)	2.90E-03
CER1	3.701 (1.666 – 8.224)	1.32E-03
HOXC8	3.625 (1.522 – 8.632)	3.63E-03
NPPC	3.571 (1.723 – 7.402)	6.21E-04
PMVK	3.545 (1.487 – 8.447)	4.29E-03
LAMTOR5	3.522 (1.727 – 7.18)	5.33E-04
NEURL1	3.434 (1.531 – 7.705)	2.77E-03
RBPJL	3.433 (1.811 – 6.507)	1.57E-04
BAG1	3.389 (1.56 – 7.358)	2.04E-03
RNASEH2C	3.388 (1.419 – 8.088)	6.00E-03
RWDD1	3.362 (1.41 – 8.017)	6.24E-03
OR8S1	3.351 (1.511 – 7.436)	2.94E-03
MRPS5	3.334 (1.399 – 7.944)	6.57E-03
PRMT1	3.316 (1.704 – 6.45)	4.15E-04
SIGLEC12	3.312 (1.618 – 6.776)	1.05E-03
WDR83OS	3.294 (1.467 – 7.398)	3.87E-03
MRPL46	3.279 (1.593 – 6.752)	1.27E-03
COX14	3.240 (1.528 – 6.869)	2.17E-03
ZNF581	3.220 (1.437 – 7.213)	4.49E-03
AUNIP	3.215 (1.819 – 5.681)	5.82E-05
PARL	3.183 (1.548 – 6.544)	1.64E-03
CTDNEP1	3.178 (1.424 – 7.096)	4.78E-03
CIB2	3.177 (1.543 – 6.54)	1.71E-03
PAF1	3.146 (1.478 – 6.697)	2.95E-03
NUDT2	3.126 (1.461 – 6.692)	3.33E-03
COQ10A	3.124 (1.218 – 8.012)	1.78E-02
B4GALT7	3.104 (1.464 – 6.582)	3.13E-03
CTNS	3.097 (1.406 – 6.823)	5.03E-03
SNX21	3.089 (1.44 – 6.628)	3.78E-03
PEG10	3.084 (1.397 – 6.812)	5.34E-03
NEIL2	3.069 (1.485 – 6.341)	2.46E-03

HR: hazard ratio

CI: confidence interval

### Final candidate molecules identified by screening

PEG10 was identified by both analyses (HR = 3.0844, 95% CI: 1.397–6.812; p-value = 0.0053). Thus, increased PEG10 expression was chosen as the candidate marker for shorter RFS in those with localized PACa with a Gleason score ≥8.

The definition of overexpression in bioinformatics analysis is complex. It is not known whether z-score ≥1 is the most appropriate definition of overexpression. Additionally, we excluded genes overexpressed in less than 5% of the population. However, this exclusion may also result in inaccurate estimations. Therefore, we investigated whether increased expression of PEG10 is linked to RFS using a different definition of increased expression: a z-score ≥1, ≥1.5, or ≥2, without the exclusion of genes overexpressed in less than 5% of the population. Again, increased expression of PEG10 was identified as a biomarker for shorter RFS when using the dataset based on localized PACa (HR = 3.0844; 95% CI, 1.397–6.812; p = 0.0053, for z-score ≥1; HR = 8.6811; 95% CI, 2.557–29.47; p = 0.0005, for z-score ≥1.5; and HR = 8.812; 95% CI, 2.01–38.63; p = 0.0039, for z-score ≥2).

### Validation of the effect of PEG10 expression on survival

**Figure 3:**
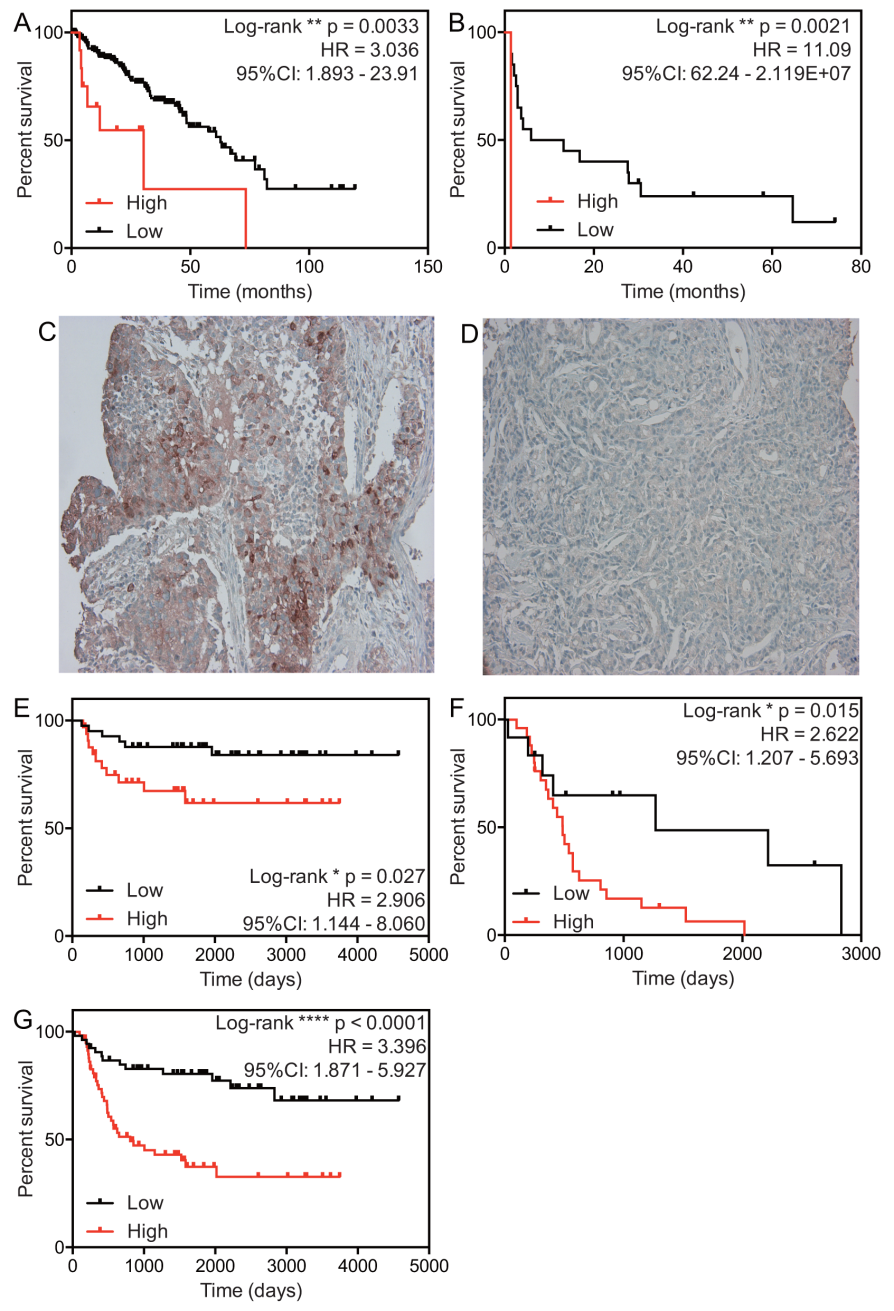
Effect of PEG10 expression on survival of PACa patients **(A** and **B)** Relapse-free survival curves for prostate cancer patients in the TCGA dataset (A) and the GSE21032 dataset (B). The definition of a high PEG10 expression is a z-score ≥1. **(C** and **D)** Representative images of PEG10 expression: high expression (C) and low expression (D). **(E–G)** Relapse-free survival curves for the clinical cohorts with localized PACa (E) and metastatic PACa (F). (G) Relapse-free survival for total PACa (G). Each population was divided into two groups (high and low expressing subpopulations), according to median expression of PEG10 protein.

We investigated the impact of PEG10 overexpression (z-score ≥1) on RFS using the TCGA dataset, different statistical models, Kaplan-Meier analysis, and the log-rank test. Overexpression of PEG10 was linked to shorter RFS (HR = 3.036; 95% CI, 1.893–23.91; p = 0.0033 [log-rank test]) (Figure 3A). The effect of PEG10 overexpression on survival was also validated in a different dataset that included both localized and metastatic cases with a Gleason score ≥8 (the GSE21032 dataset) (Table 4) [10]. Again, PEG10 overexpression was linked to shorter RFS (HR = 11.09; 95% CI, 62.24–2.119E+07; p = 0.0021) (Figure 3B).

**Table 4:** Baseline characteristics of GSE21032 database (Gleason score ≥ 8)

Total cases	21
Age	
Average	59.7
Range	46-71
T category	
T2a	1
T2b	1
T2c	1
T3a	6
T3b	7
T3c	2
T4	3
Gleason score	
3+5	1
4+4	8
5+3	2
4+5	10

To investigate whether expression of PEG10 protein in biopsy samples taken at the time of initial diagnosis was also linked to RFS of those with localized or metastatic PACa, we collected PACa biopsy samples from patients diagnosed at St. Marianna University Hospital between 2003 and 2014 (Table 5). Increased protein expression of PEG10 in clinical samples from patients with localized PACa was linked to shorter RFS (HR = 2.906; 95% CI, 1.144–8.060; p = 0.027) (Figure 3C–3E). Increased protein expression of PEG10 was also linked to shorter RFS of patients with metastatic PACa (HR = 2.62; 95% CI, 1.207–5.693; p = 0.015) (Figure 3F). The same was true when we examined total (local plus metastatic) PACa (HR = 3.396; 95% CI, 1.871–5.927; p < 0.0001) (Figure 3G). Furthermore, the effect of PEG10 expression remained significant in multivariate analysis using a Cox proportional hazard model adjusted for age, initial PSA level, main treatment (hormonal therapy or others), and stage (Table 6).

**Table 5:** Baseline characteristics of clinical samples

Age	Median range	72.055 - 86
T category	T2a	6
	T2b	30
	T2c	36
	T3a	16
	T3b	11
	T4	6
	NA	7
Gleason score	3 + 5	4
	5 + 3	3
	4 + 4	55
	4 + 5	29
	5 + 4	14
	5 + 5	7
Histology	Adenocarcinoma NOS	112
Treatment	RP	29
	RT	45
	HT	38

NA: not available

RP: radical prostatectomy

RT: radiation therapy

HT: hormonal therapy

**Table 6:** Multivariate analysis of PEG10 expression on RFS

	Unadjusted		Adjusted	
	HR (95%CI)	p-value	HR (95%CI)	p-value
PEG10	3.51 (1.84-6.70)	^***^ 1.44E-04	2.50 (1.27-4.93)	^**^ 8.11E-03
Age	0.99 (0.95-1.04)	6.79E-01	9.71 (0.92-1.02)	2.44E-01
iPSA	1.00 (1.00-1.00)	^*^ 2.40E-02	1.00 (0.99-1.00)	8.82E-01
Treatment	6.37 (3.44-11.77)	^***^ 3.58E-09	1.51 (0.27-844.23)	1.87E-01
Stage	2.49 (1.80-3.46)	^***^ 4.97E-08	0.61 (0.08-4.80)	6.52E-01

HR: hazard ratio

CI: confidence interval

iPSA: initial PSA

Treatment includes localized treatment (radical prostatectomy and radiation therapy) and hormonal therapy.

Age, iPSA and stage were evaluated as a continuous variable.

### Link between PEG10 expression and neuroendocrine differentiation

**Figure 4:**
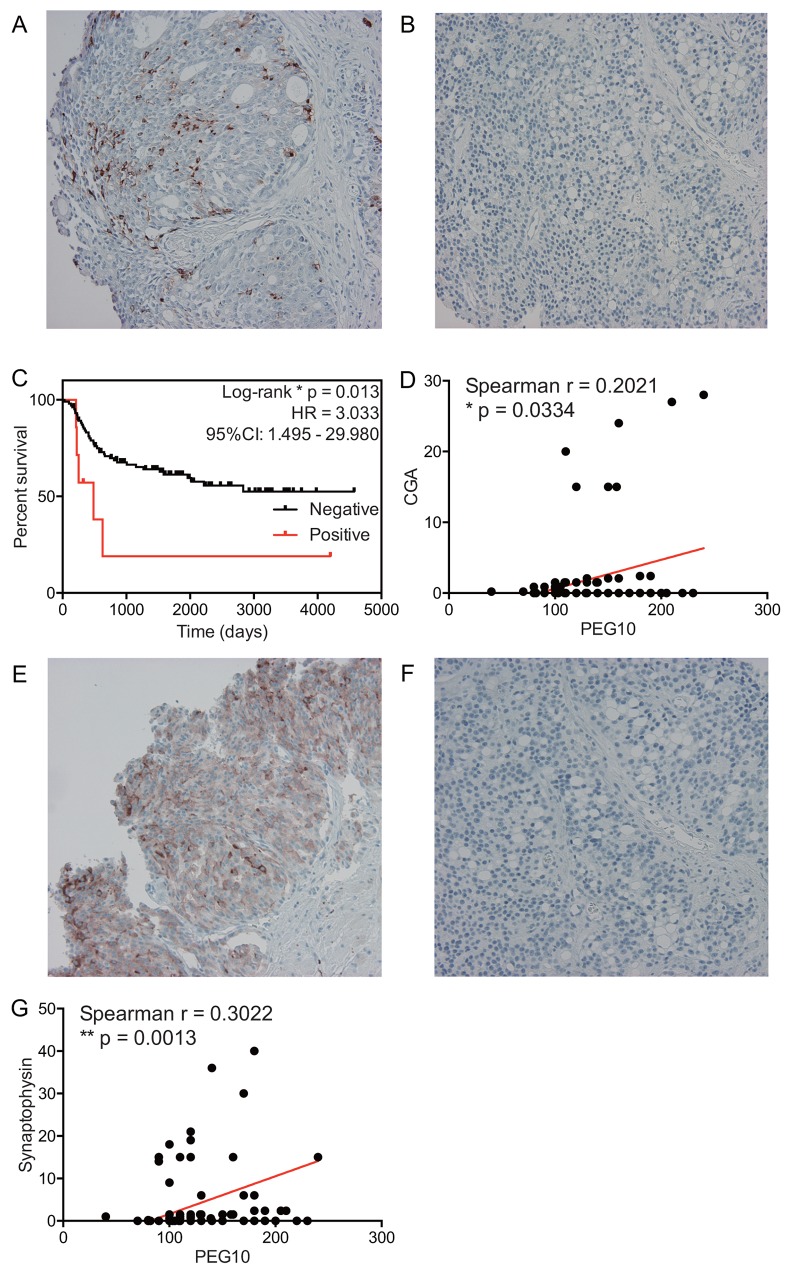
A link between PEG10 expression and neuroendocrine differentiation **(A and B)** Representative images showing CGA expression: high (A) and low (B). **(C)** Relapse-free survival curves for the clinical cohort with localized PACa. The definition of high CGA expression is a CGA-positive population ≥1%. **(D)** Dot plot showing the correlation between PEG10 expression and CGA expression. **(E and F)** Representative images showing synaptophysin expression: high (E) and low (F). **(G)** Dot plot showing the correlation between PEG10 expression and synaptophysin expression.

A recent report suggests that PEG10 expression is increased in PACa with focal neuroendocrine differentiation [9]. Thus, we stained clinical samples with chromogranin A (CGA), a marker for neuroendocrine differentiation [11], to explore the link between increased PEG10 expression and neuroendocrine differentiation. Because expression of CGA in PACa tissue is negligible, the effect of CGA expression on clinical outcome is a matter for debate [12, 13]. Recent studies suggest that a scoring system based on the population of CGA-positive cells (0: <1% positive cells; 1: 1–10% positive cells; 2: >10% positive cells) in biopsy samples taken at the time of initial diagnosis shows a direct link with clinical outcome [13-15]. Therefore, we stained samples for CGA and examined the relationship between CGA expression at the time of diagnosis and clinical outcome. We found that increased expression of CGA was indeed linked to shorter RFS (Figure 4A–4C). Additionally, we assessed the association between PEG10 expression and CGA expression using the H-score. Interestingly, increased expression of PEG10 was positively associated with expression of CGA (Spearman r = 0.2021; p = 0.0334) (Figure 4D). Furthermore, expression of PEG10 was positively associated with expression of synaptophysin, another representative marker of neuroendocrine differentiation (Figure 4E–4G). This suggests that increased expression of PEG10 at the time of initial diagnosis may predict neuroendocrine differentiation.

## DISCUSSION

To apply biomarker-driven cancer therapy to PACa, we developed a method called bioinformatics-to-clinic sequential analysis. A bioinformatic approach can be a useful tool; however, it has several limitations. One such limitation is that data that fit the study model perfectly are often not available. In the current study, the dataset required for our model needed to include not only localized, but also metastatic, PACa. A dataset including both localized and metastatic PACa was available, but it contained only 21 cases. Thus, we used a TCGA dataset that included only localized PACa; however, the sample number (201 cases) was acceptable for bioinformatics analysis. Next, we used the clinical samples that included both localized and metastatic PACa to ascertain whether biomarkers of a poor prognosis identified by the bioinformatics approach (based on the localized PACa dataset) were applicable to the clinical samples from patients with localized or metastatic PACa.

When identifying DEGs (the first step in the *in silico* analysis), the definition of “shorter survival” is complex. To identify DEGs in this study, we divided the dataset according to 1, 3, or 5 year survival. It is still not known if these criteria are the most appropriate. This is an important consideration because extraction of genes that emerge in the differential settings may avoid under- or over-diagnosis. Another issue related to DEGs is that DEGs are identified by a comparison of the average value for each group. If a particular gene has a strong link to survival when overexpressed, but overexpression in the population is low, the average value of the gene’s expression in a cohort of shorter survival will not be high. Thus, identification of DEGs may not always give the most accurate estimation of genes relevant to survival time. The second step, a calculation of HR, may also be problematic. In this study, we calculated HR in several definitions of increased expression. Even in the different settings, increased expression of PEG10 was linked to shorter survival. However, it is still not known which definition is appropriate. As described above, there are problems with *in silico* analysis; therefore, to compensate for these we developed the bioinformatics-to-clinic sequential analysis approach to check for concordance between different analytical procedures. Indeed, our results indicate that our approach is reliable and provides a high-quality outcome.

The transcription factor c-Myc induces production of PEG10 mRNA [16]. Due to the instability of c-Myc mRNA, it is not possible to investigate the relationship between c-Myc mRNA and PEG10 mRNA expression using bioinformatics [16]. However, if overexpression of PEG10 is associated with up-regulation of c-Myc in PACa, c-Myc inhibition may be a therapeutic option for the subset of PACa patients with a poor prognosis and overexpression of PEG10. Currently, several c-Myc inhibitors are being tested in clinical trials [17]. The present study may indicate an additional therapeutic application for c-Myc inhibitors in a subset of PACa patients that have a poor prognosis, and possibly in patients with neuroendocrine prostate cancer.

## MATERIALS AND METHODS

### Bioinformatic analysis

Gene count data from PACa TCGA samples (RNA sequencing) were downloaded from the Genomic Data Commons Data Portal (https://gdc-portal.nci.nih.gov). To identify DEGs, raw counts were entered into the TCC package in R [18]. The definition of DEGs is a false discovery rate (FDR) of <0.05 and a logFC ≥1 or ≤-1. Z-scores (RNA seq V2 RSEM) available in cBioPortal (http://www.cbioportal.org) were used to calculate the HR [19]. Overexpression was defined as a z-score ≥1, ≥1.5 or ≥2. The HR of genes was calculated using Cox proportional hazard models and the coxph function located in the survival library in R.

### Statistical analysis

HRs were calculated using the Cox proportional hazard model, and significance was assessed using the log-rank test. The Kaplan-Meier method was used for survival analysis. The link between PEG10 and CGA or synaptophysin expression was assessed using the Spearman’s Rank-Order Correlation test. For multivariate analysis, Cox proportional hazard models were performed using the coxph function in the survival library in R. All other analyses were performed with GraphPad prism. Differences were considered statistically significant when the two-tailed p-value was <0.05.

### Patients

Patients diagnosed with PACa by biopsy at St. Marianna University Hospital from January 2003 to August 2014 were considered for inclusion in this study. Patients without exact information regarding their case and patients without standard therapy due to advanced age or severe comorbidities were excluded. Of the remaining patients, 121 had a Gleason score ≥8 and became the cohort assessed in this study.

### Antibodies

The following antibodies were used in this study: anti-PEG10 (GeneTex: 4C10A7, 1:400); anti-chromogranin A (Dako: N1535, 1:10), and anti-synaptophysin (Nichirei Bioscience: 413831, 1:2).

### Immunohistochemistry (IHC) and measurement of protein expression

Immunohistochemistry (IHC) was performed as described previously [20]. Briefly, for PEG10 and synaptophysin staining, antigen retrieval was performed using Antigen Retrieval Solution (pH 9.0) (Nichirei bioscience) at 95°C in a steamer for 40 min, followed by incubation with an anti-PEG10 or an anti-synaptophysin antibody for 60 min. For CGA staining, the sections were incubated with an anti-CGA antibody for 60 min without antigen retrieval. For protein expression determinations, more than 500 cells in five high-resolution fields were evaluated and their H-score was calculated. Increased expression was defined as above median.

### Study approval

Experiments with clinical samples were approved by the St. Marianna University clinical ethics committee, and all participants gave informed consent (approval number: 3181).

## SUPPLEMENTARY MATERIALS TABLE





## Abbreviations

PACa: prostate adenocarcinoma
PSA: prostate-specific antigen
CRPC: castration-resistant prostate cancer
TCGA: The Cancer Genome Atlas
DEGs: differentially expressed genes
RFS: relapse-free survival
HR: hazard ratio
FDR: false discovery rate
logFC: logarithmic fold change
CGA: chromogranin A
IHC: immunohistochemistry

## References

[1] Druker BJ, Guilhot F, O'Brien SG, Gathmann I, Kantarjian H, Gattermann N, Deininger MW, Silver RT, Goldman JM, Stone RM, Cervantes F, Hochhaus A, Powell BL (2006). Five-year follow-up of patients receiving imatinib for chronic myeloid leukemia. N Engl J Med.

[2] Dawood S, Broglio K, Buzdar AU, Hortobagyi GN, Giordano SH (2010). Prognosis of women with metastatic breast cancer by HER2 status and trastuzumab treatment: an institutional-based review. J Clin Oncol.

[3] Siegel RL, Miller KD, Jemal A (2016). Cancer statistics, 2016. CA Cancer J Clin.

[4] Andren O, Fall K, Franzen L, Andersson SO, Johansson JE, Rubin MA (2006). How well does the Gleason score predict prostate cancer death? A 20-year followup of a population based cohort in Sweden. J Urol.

[5] Epstein JI, Allsbrook WC, Amin MB, Egevad LL, Committee IG (2005). The 2005 International Society of Urological Pathology (ISUP) Consensus Conference on Gleason Grading of Prostatic Carcinoma. Am J Surg Pathol.

[6] Filson CP, Marks LS, Litwin MS (2015). Expectant management for men with early stage prostate cancer. CA Cancer J Clin.

[7] Eisenberger MA, Simon R, O'Dwyer PJ, Wittes RE, Friedman MA (1985). A reevaluation of nonhormonal cytotoxic chemotherapy in the treatment of prostatic carcinoma. J Clin Oncol.

[8] Barbieri CE, Chinnaiyan AM, Lerner SP, Swanton C, Rubin MA (2017). The Emergence of Precision Urologic Oncology: A Collaborative Review on Biomarker-driven Therapeutics. Eur Urol.

[9] Akamatsu S, Wyatt AW, Lin D, Lysakowski S, Zhang F, Kim S, Tse C, Wang K, Mo F, Haegert A, Brahmbhatt S, Bell R, Adomat H (2015). The Placental Gene PEG10 Promotes Progression of Neuroendocrine Prostate Cancer. Cell Rep.

[10] Taylor BS, Schultz N, Hieronymus H, Gopalan A, Xiao Y, Carver BS, Arora VK, Kaushik P, Cerami E, Reva B, Antipin Y, Mitsiades N, Landers T (2010). Integrative genomic profiling of human prostate cancer. Cancer Cell.

[11] Tricoli JV, Schoenfeldt M, Conley BA (2004). Detection of prostate cancer and predicting progression: current and future diagnostic markers. Clin Cancer Res.

[12] Bostwick DG, Qian J, Pacelli A, Zincke H, Blute M, Bergstralh EJ, Slezak JM, Cheng L (2002). Neuroendocrine expression in node positive prostate cancer: correlation with systemic progression and patient survival. J Urol.

[13] Theodorescu D, Broder SR, Boyd JC, Mills SE, Frierson HF (1997). Cathepsin D and chromogranin A as predictors of long term disease specific survival after radical prostatectomy for localized carcinoma of the prostate. Cancer.

[14] Krauss DJ, Amin M, Stone B, Ye H, Hayek S, Cotant M, Hafron J, Brabbins DS (2014). Chromogranin A staining as a prognostic variable in newly diagnosed Gleason score 7-10 prostate cancer treated with definitive radiotherapy. Prostate.

[15] Krauss DJ, Hayek S, Amin M, Ye H, Kestin LL, Zadora S, Vicini FA, Cotant M, Brabbins DS, Ghilezan MI, Gustafson GS, Martinez AA (2011). Prognostic significance of neuroendocrine differentiation in patients with Gleason score 8-10 prostate cancer treated with primary radiotherapy. Int J Radiat Oncol Biol Phys.

[16] Li CM, Margolin AA, Salas M, Memeo L, Mansukhani M, Hibshoosh H, Szabolcs M, Klinakis A, Tycko B (2006). PEG10 is a c-MYC target gene in cancer cells. Cancer Res.

[17] Chen BJ, Wu YL, Tanaka Y, Zhang W (2014). Small molecules targeting c-Myc oncogene: promising anti-cancer therapeutics. Int J Biol Sci.

[18] Sun J, Nishiyama T, Shimizu K, Kadota K (2013). TCC: an R package for comparing tag count data with robust normalization strategies. BMC Bioinformatics.

[19] Bourgon R, Gentleman R, Huber W (2010). Independent filtering increases detection power for high-throughput experiments. Proc Natl Acad Sci U S A.

[20] Nagasawa S, Sedukhina AS, Nakagawa Y, Maeda I, Kubota M, Ohnuma S, Tsugawa K, Ohta T, Roche-Molina M, Bernal JA, Narvaez AJ, Jeyasekharan AD, Sato K (2015). LSD1 Overexpression Is Associated with Poor Prognosis in Basal-Like Breast Cancer, and Sensitivity to PARP Inhibition. PLoS One.

