# Supplementary material for: A bioinformatics-to-clinic sequential approach to analysis of prostate cancer biomarkers using TCGA datasets and clinical samples: a new method for precision oncology?
Source: Oncotarget. 2017 Aug 24;8(59):99601–11. doi: 10.18632/oncotarget.20448 (PMC5725118; doi:10.18632/oncotarget.20448)
Supplement: Supplementary file 1 [file oncotarget-08-99601-s001.pdf]

A bioinformatics-to-clinic sequential approach to analysis of prostate cancer biomarkers using TCGA datasets and clinical samples: a new method for precision oncology?

SUPPLEMENTARY MATERIALS

Supplementary Table 1: Baseline characteristics of TCGA and GSE21032 database. Baseline characteristics of whole cohort (Gleason score ≥8) (ALL in the bottom tab) and cohort divided at 1, 3 and 5 year survival (1year, 3year and 5year in the bottom tab, respectively) in TCGA and whole cohort of GSE321032 (GSE21032 in the bottom tab) are shown.

See Supplementary File 1
